# Stage-specific, Nonlinear Surface Ozone Damage to Rice Production in China

**DOI:** 10.1038/srep44224

**Published:** 2017-03-13

**Authors:** Colin A. Carter, Xiaomeng Cui, Aijun Ding, Dalia Ghanem, Fei Jiang, Fujin Yi, Funing Zhong

**Affiliations:** 1 University of California, Davis, CA, USA; 2 Nanjing University, Jiangsu, China; 3 Nanjing Agricultural University, Jiangsu, China.

## Abstract

China is one of the most heavily polluted nations and is also the largest agricultural producer. There are relatively few studies measuring the effects of pollution on crop yields in China, and most are based on experiments or simulation methods. We use observational data to study the impact of increased air pollution (surface ozone) on rice yields in Southeast China. We examine nonlinearities in the relationship between rice yields and ozone concentrations and find that an additional day with a maximum ozone concentration greater than 120 ppb is associated with a yield loss of 1.12% ± 0.83% relative to a day with maximum ozone concentration less than 60 ppb. We find that increases in mean ozone concentrations, SUM60, and AOT40 during panicle formation are associated with statistically significant yield losses, whereas such increases before and after panicle formation are not. We conclude that heightened surface ozone levels will potentially lead to reductions in rice yields that are large enough to have implications for the global rice market.

Ozone air pollution threatens future global food security^1^,^2^,^3^,^4^. For certain regions and crops, including rice in China, ozone pollution is projected to be more damaging to food production than climate change^5^. Air pollution has been increasing at an alarming rate in China^6^, a country that is the world’s largest rice producer and importer^7^. Hence, any yield reduction has repercussions for rice prices and global food security. Ozone regulation could improve rice production in China. However, to design such policies effectively, a better understanding of the complex relationship between ozone and rice yields is needed. Here we examine this relationship using county-level longitudinal data from Southeast China. We find that increases in mean ozone concentrations, SUM60, and AOT40 during panicle formation (PF) are associated with statistically significant yield loss, whereas such increases before and after PF are not. We further examine the effect of “peak” ozone days. We find that an additional day with a maximum ozone concentration greater than 120 ppb (parts per billion) is associated with a yield loss of 1.12% ± 0.83% relative to a day with maximum ozone concentration less than 60 ppb. This evidence on nonlinearities in the relationship between rice yields and ozone pollution should inform ozone regulation policies and open new directions for future research.

Rice is the most important crop in China. In 2015, China harvested about 30% of the global production of rice, 206.4 million metric tons (*mmt*) of rough rice^8^. Although China maintains a policy target of 95% self-sufficiency in rice^9^, China is now the world’s largest rice importer with imports reaching 6.2 *mmt* (rough equivalent) in 2015 and China is expected to remain the largest rice importer for the next decade^10^ (Supplementary Figure 1). Only 8% of global production of rice is traded internationally in an average year, which means the world price is very sensitive to small trade volume changes.

China has some of the worst and continuously rising air pollution on the globe^11^,^12^,^13^,^14^. Current evidence on yield response of rice to heightened ozone concentrations is based primarily on controlled experiments^15^,^16^,^17^. Previous studies using historical data have examined the effect of ozone pollution on a number of crops in the United States and India, but only for winter wheat in China^18^,^19^,^20^,^21^,^22^,^23^.

This study quantifies the effect of heightened surface ozone on rice yields in China. We perform a county-level longitudinal analysis for five provinces in Southeast China. Our data spans three years, 2006, 2008 and 2010. Because there are no widespread rural station-monitored ozone records in China, we use hourly surface ozone concentrations simulated by the Community Multi-scale Air Quality Model (CMAQ), which is well-validated and was used in previous work^23^. The spatial distribution of simple average ozone concentrations is illustrated in Fig. 1. Figure 2 presents the average daily ozone concentration over the growing season for our sample. We estimate a multivariable model of rice yields that includes surface ozone concentrations as well as weather variables, fertilizer use, and natural disasters. Our model includes county and year fixed effects, controlling for county-specific or year-specific unobservable factors. Hence, our analysis is robust to omitted variables that are either county-specific or year-specific. Using this model, we examine two important questions: First, is ozone damage to rice yield heterogeneous during different stages of the growing season? Second, how do “peak” ozone days affect yield? To answer these questions, we specifically examine yield loss associated with heightened ozone concentrations during PF, an important stage of the growing season for yield^24^,^25^,^26^.

Panel A of Fig. 3 presents the estimated yield loss associated with a one-unit increase in 8-hour simple average ozone concentration, SUM60 and AOT40 before, during and after PF. For all the measures we consider, we find no statistically significant evidence that heightened ozone concentrations before and after PF are associated with yield loss. We find that a one ppb increase in the 8-hour simple average ozone measure during PF is associated with an 0.48% ± 0.42% reduction in yield. For SUM60 and AOT40, an additional ppmh (parts per million hours) during PF is associated with an 0.74% ± 0.50% and 1.59% ± 1.14% reduction in annual yield, respectively. Additional details on the estimated yield loss due to a unit increase in the above pollution measures during the entire growing season are in Supplementary Methods VI.

The above results provide strong statistical evidence that PF is the critical stage of plant growth in terms of ozone damage to rice yield. To contextualize how important these estimates are, we present the distribution of within-county year-to-year variation of simple average, SUM60 and AOT40 during PF in Panel B of Fig. 3. In more than 45% of within-county changes in our sample, we observe more than 4 ppb year-to-year changes in simple average, more than 3 ppmh changes in SUM60, and more than 1.5 ppmh changes in AOT40. According to our results, these changes would be associated with changes in yield of more than 1.92%, 2.22% and 2.40%, respectively.

We next turn to the question of the effect of “peak” ozone days. We sort days into 20 ppb categories according to the 8-hour maximum ozone concentration in that day (<60, 60–80, 80–100, 100–120, >120 ppb). Panel A in Fig. 4 shows the effect of an additional day with an 8-hour maximum ozone concentration in the different categories relative to a day with an 8-hour maximum less than 60 ppb. We find that an additional day with an 8-hour maximum ozone concentration between 100–120 ppb is associated with 0.94% ± 0.43% yield loss, whereas an additional day with an 8-hour maximum ozone concentration above 120 ppb is associated with 1.28% ± 0.86% yield loss. Panel B in Fig. 4 illustrates the proportion of days that fall into the different categories. About 7% of the days during PF in our sample have an 8-hour maximum ozone concentration above 100 ppb.

To summarize our results, a roughly 1% rice yield loss is associated with the following increases in the different ozone measures we consider during PF: 2.08 ppb in simple average ozone concentrations, 1.35 ppmh in SUM60, 0.63 ppmh in AOT40, and an additional day with extreme ozone concentrations above 100 ppb. For the five provinces we consider, our results suggest that this change in yield would result in a loss of 337 thousand *mt* of paddy rice output using 2010 production levels (Supplementary Table 1). If this yield loss occurred in all of China, it would lead to a loss of about 2 *mmt* of annual rice output, about one third of China’s current annual rice imports.

Ozone regulation is clearly an important food-security policy tool for China, with implications for the rest of the world. This study points to the complex relationship between rice yields and ozone pollution. Our results confirm that the gains from ozone regulation for rice in China are large, similar to previous controlled experiments^16^,^27^ (Supplementary Methods V and VI). Our contribution lies in providing strong statistical evidence that the gains from ozone regulation are nonlinear and specific to stages of plant growth. Our results suggest that the largest yield gains from ozone regulation occur during PF. Furthermore, days with 8-hour maximum ozone concentration larger than 100 ppb during PF are most harmful to yield. Our study points to an important direction for future research, examining the heterogeneity in crop response to heightened ozone during different stages of plant growth as well as to days with “peak” ozone concentrations. Future controlled experiments as well as longitudinal studies in different countries are required to further examine these aspects of heterogeneity in crop response to surface ozone, which are key to designing ozone regulation policies that reduce threats to global food security.

## Methods

The baseline statistical model is a standard linear fixed effects model of the natural logarithm of yield. The results in Panel A of Fig. 3 are obtained from the following regression model. Let *i* denote a county, *r* denote a province, and *t* denote a year,


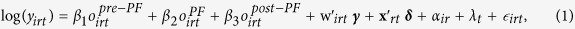


where 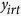 denotes the annual rice yield for county *i* in province *r* in year *t*, **w**_*irt*_ is a vector of weather variables, including degree days and total precipitation in the entire growing season, **x**_rt_ is a vector of control variables at the provincial level including fertilizer application on rice and natural disaster variables, *α*_*ir*_ and *λ*_*t*_ control for county-specific and year-specific unobservables, respectively, and *∈*_*irt*_ represents the error term. 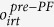, 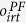 and 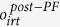 are O_3_ measures before, during and after PF, respectively. Details on the data and variable construction are in Supplementary Methods I and III, respectively.

**Figure 2:**
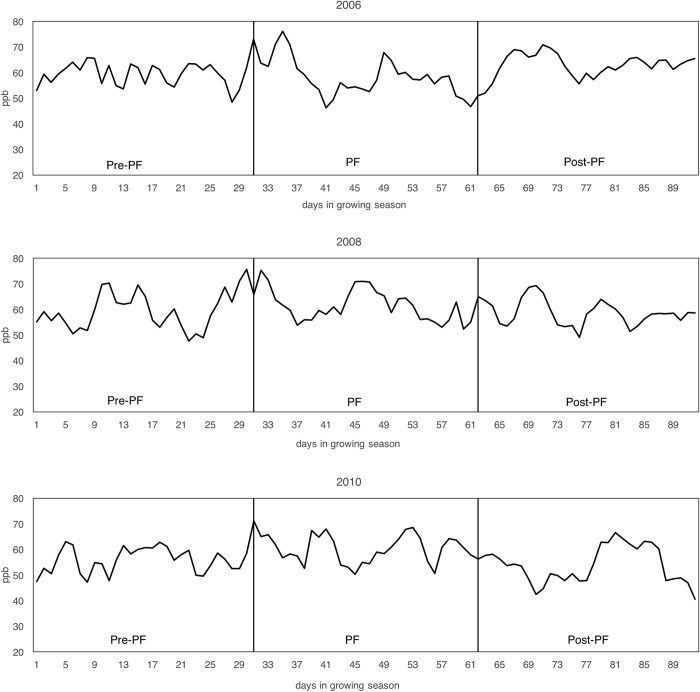
Average Daily Daytime Ozone Levels over the Growing Season. Daily 8-hour (10 am-5 pm) average ozone concentrations are averaged across the two growing seasons for all counties for each day in a year. We limit our plot of post-PF to the one-month period right after PF for simplicity. The actual post-PF period in the second growing season for counties in Guangdong and Hainan extends two more weeks (for details see Supplementary Table 2).

**Figure 3:**
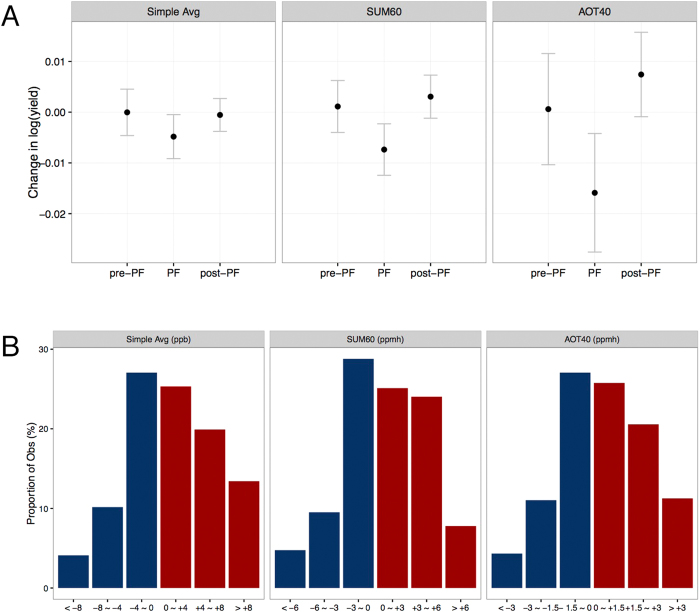
Yield Loss Associated with a Unit Increase in Simple Average, SUM60 and AOT40 Ozone Measures During Different Stages of the Growing Season. **(A)** Ozone coefficients. Estimates are obtained from the regressions for simple average, SUM60 and AOT40 constructed separately over three periods: the period before PF (Pre-PF), the PF period (PF), and the period after PF (Post-PF), given in equation (1). Black dots represent point estimates, and gray bars represent 95% confidence intervals based on prefecture-level cluster-robust standard errors (refer to Section II in Supplementary Methods for the relationship between prefectures and counties). The regression includes control variables for weather, fertilizer application, and natural disasters, as well as county and year fixed effects. **(B)** Distribution of within-county ozone variation during PF. We report the histograms for within-county changes in simple average, SUM60 and AOT40 during PF. For each variable, observations are categorized into six bins according to year-to-year variation. The range of the bins is labeled under each bar.

The simple average O_3_ is measured in ppb and equals the mean of the hourly O_3_ concentrations for 10 am-5 pm during the different stages of the growing season. We perform sensitivity analyses to verify that our estimates are robust to the definition of the daytime window (Supplementary Methods V). SUM60 and AOT40 measure the cumulative O_3_ above a certain threshold, and the unit of measure is ppmh. SUM60 sums up the hourly average O_3_ concentrations greater or equal to 60 ppb for different stages in the growing season. AOT40 uses an alternative threshold of 40 ppb, and sums the “excess” hourly O_3_ concentrations above 40. Figure 2 shows spatial and temporal variation of simple average O_3_ during PF, and variation of SUM60 and AOT40 can be found in Supplementary Methods. Formulas for the ozone, weather, fertilizer, and natural disaster variables can be found in Supplementary Methods III.

**Figure 4:**
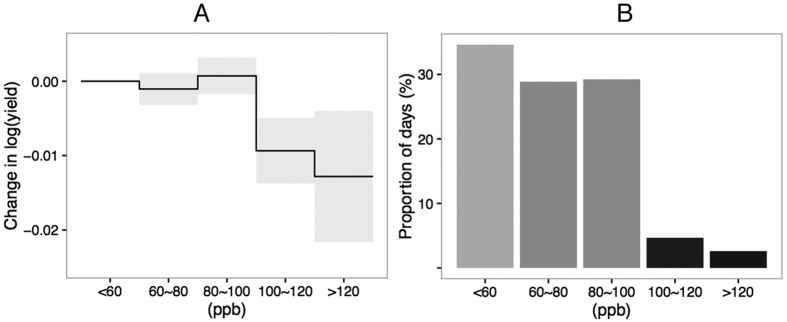
Yield Loss Associated with an Additional Day with 8-hour Maximum Ozone Concentration in 20 ppb Categories Above 60 ppb Relative to a Day with an 8-hour Maximum Ozone Concentration of <60 ppb. **(A)** Ozone coefficients obtained from regression equation (2). The gray band represents the 95% confidence interval for each coefficient from the regression based on prefecture-level cluster-robust standard errors (refer to Section II in Supplementary Methods for the relationship between prefectures and counties). **(B)** The proportions of days with the daily maximum ozone concentration falling into different bins. The range of the bin is labeled under each bar.

The following model is used to obtain the results in Panel A in Fig. 4


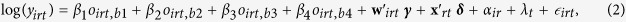


where *o*_*irt, bj*_ is the number of days in the *j*^*th*^ category during PF for *j* = 1, 2, 3, 4. The categories are determined by the maximum 8-hour ozone concentration in a day in the following ppb bins {60–80, 80–100, 100–120, >120}, where the reference category is <60 ppb. For a formal definition of *o*_*irt, bj*_, see Supplementary Methods IV.

## Additional Information

**How to cite this article:** Carter, C. A. *et al*. Stage-specific, Nonlinear Surface Ozone Damage to Rice Production in China. *Sci. Rep.* **7**, 44224; doi: 10.1038/srep44224 (2017).

**Publisher's note:** Springer Nature remains neutral with regard to jurisdictional claims in published maps and institutional affiliations.

## Supplementary Material

Supplementary Methods

## Figures and Tables

**Figure 1:**
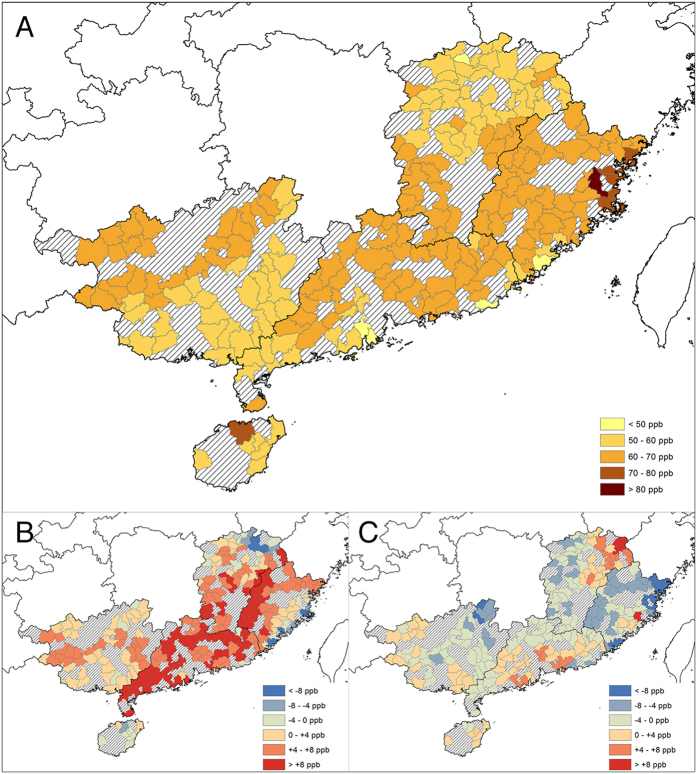
Levels and Variations of Simple Average Ozone during PF. **(A**) Simple average ozone during PF averaged across 2006, 2008, and 2010. **(B)** Year-to-year variation of simple average ozone during PF from 2006 to 2008. **(C)** Year-to-year variation of simple average ozone during PF from 2008 to 2010. Counties shown in color are used in the regression analysis. Black and white shaded areas are not included because they did not plant rice consistently over the time period analyzed. Regions shown in white are outside the five provinces in our sample. These maps were generated by mapping calculated values from our data to county polygons by using ArcGIS 10 (http://www.esri.com/software/arcgis). The shapefile of polygons was obtained from Global Administrative Areas (http://gadm.org).
