# Supplementary material for: Stage-specific, Nonlinear Surface Ozone Damage to Rice Production in China
Source: Sci Rep. 2017 Mar 13;7:44224. doi: 10.1038/srep44224 (PMC5346997; doi:10.1038/srep44224)
Supplement: Supplementary Methods [file srep44224-s1.pdf]

Stage-specific, Nonlinear Surface Ozone Damage to Rice Production in China: Supplementary Methods

Colin A. Carter, Xiaomeng Cui, Aijun Ding, Dalia Ghanem, Fei Jiang, Fujin Yi, and Funing Zhong *

I. Data

The rice production data were obtained from the Institute of Agricultural Information at the Chinese Academy of Agricultural Sciences. The data include county-level annual rice output and planted area in 2006, 2008, and 2010. The hourly O_3 data are simulated using the Community Multi-scale Air Quality Model (CMAQ) and mapped to the county level. We obtained station-level weather data from the China Meteorological Data Sharing Service System to construct weather variables. Fertilizer application was sourced from the China Agricultural Product Cost and Revenue Yearbook. National Data published by the National Bureau of Statistics of China provided annual natural disaster data.

II. Administrative Divisions in China

The local administration in China can be described as a top-down system with four layers – provinces, prefectures, counties, and villages. A province usually contains several prefectures, and each prefecture contains several counties, with several villages under each county's governance. Our study uses county as the smallest observational unit. We focus on five provinces in southeastern China, namely Guangxi, Guangdong, Fujian, Jiangxi, and Hainan. The panel data set we construct contains 231 counties located in 53 prefectures in the five provinces.

Most rice is double-cropped in the five provinces. According to the National Bureau of Statistics, the proportions of double-cropped rice for Fujian, Guangxi, and Jiangxi are 45.10%, 92.83%, and 86.06%, respectively; Guangdong and Hainan only plant double-cropped rice. Because we do not have information of cropping patterns at the county level, we treat all observations in our merged data as double croppers. The growing season for single rice in Fujian, Guangxi, and Jiangxi virtually overlaps with the late growing season in those provinces, which makes our definition of growing stages valid even if a county is not a double-cropper.

*Carter: Univ. of California, Davis, cacarter@ucdavis.edu. Cui: Univ. of California, Davis, ecui@ucdavis.edu. Ding: Nanjing Univ., dingaj@nju.edu.cn. Ghanem: Univ. of California, Davis, dghanem@ucdavis.edu. Jiang: Nanjing Univ., jiangf@nju.edu.cn. Yi: Nanjing Agricultural Univ., fujinyi@njau.edu.cn. Zhong: Nanjing Agricultural Univ., fnzhong@njau.edu.cn

III. Empirical approach

We use a multivariate panel model with county and year fixed effects to measure the impact of O_3 on rice yield. The general form of this model is given by

$$\log(y_{irt}) = \mathbf{o}'_{irt}\beta + \mathbf{w}'_{irt}\gamma + \mathbf{x}'_{rt}\delta + \alpha_{ir} + \lambda_t + \varepsilon_{irt}, \tag{1}$$

where y_{irt} represents the average rice yield for county i in province r in year t, $\mathbf{o}_{irt}$ is a vector of ozone measures, $\mathbf{w}_{irt}$ is a vector of environmental factors, $\mathbf{x}_{rt}$ is a vector of additional control variables at the province level, including fertilizer application and natural disasters, where r indexes provinces, α_{ir} represents county fixed effects that control for time-invariant characteristics of county i, λ_t represents year fixed effects that capture general changes across years like technological advances and policy changes, ε_{irt} is the error term. The standard errors are clustered at the prefecture level.

Rice yields

The planted area is the sum of planted areas for the early and late growing seasons. The units of measure are hectares (ha) for the planted area and metric tons (mt) for output. We calculate the average yield by dividing the annual total output by the annual total planted area. Yields are measured in mt per ha. Observations with yields above 20 mt per ha were dropped from the dataset because they are likely reporting errors. The data contain original observations for 315 counties. Among them, 279 counties were successfully merged with our other data. Among the merged counties, 231 counties display non-zero planted areas in all three years. This sample corresponds to about 20% of China's rice planted area and 18% of its rice output (Table 1).

Ozone measures

We construct three different O_3 measures for our baseline regression: simple average, SUM60, and AOT40. In line with the literature, we construct these ozone measures based on an 8-hour time window (10 am - 5 pm) each day. We also perform sensitivity analyses with various time windows.

For our main regression, the simple average measure is defined as

$$o_{irt,m} = \frac{\sum_{d\in\mathbf{D}}}{\bar{d}} \frac{\sum_{h\in[10,17]}}{8} \left\{ oc_{irt,d,h} \right\}. \tag{2}$$

The SUM60 measure is defined as

$$o_{irt,s} = \frac{1}{1000} \sum_{d\in\mathbf{D}} \left\{ \sum_{h\in[10,17]} \left\{ oc_{irt,d,h} \middle| oc_{irt,d,h} \geq 60 \right\} \right\}. \tag{3}$$

The AOT40 measure is defined as

$$o_{irt,a} = \frac{1}{1000} \sum_{d\in\mathbf{D}} \left\{ \sum_{h\in[10,17]} \max\{(oc_{irt,d,h} - 40), 0\} \right\}. \tag{4}$$

In the equations above, $oc_{irt,d,h}$ represents the O_3 concentration (measured in ppb) for county i in province r during hour h of day d in year t, where $h = 1, 2, ..., 24$ and $d \in \mathbf{D}$. $\mathbf{D}$ denotes the set

of days corresponding to the period that we construct the ozone measure over, and $\bar{d}$ denotes the maximum number of days in that period.

Scientific evidence suggests rice is most sensitive to O_3 pollution during panicle formation (PF)[1–3]. To measure this statistically, we divide the growing season into three periods: before, during, and after panicle formation (labeled as pre-PF, PF, and post-PF, respectively). Table 2 shows the dates of these periods for the different provinces in our data set. These dates are determined according to the rice progress report by the National Bureau of Statistics of China. Because we do not observe rice yields specific to the early and late seasons, we create pre-PF, PF, and post-PF measures that average over the two growing seasons. This only imposes the assumption that the marginal effects in the early and the late season are equal, which is arguably valid because the crop variety typically does not differ among the early and late seasons.

We construct three variables for each ozone measure over the three subperiods and label them using superscripts, i.e., $\mathbf{o}_{irt} = (o_{irt}^{pre-PF}, o_{irt}^{PF}, o_{irt}^{post-PF})'$ in equation (1).

Weather variables

The weather data contain daily precipitation, and daily maximum, minimum, and average temperature. The county-level data were compiled from the station-level as follows. First, the inverse distance weighted (IDW) method was used to interpolate the climate data at 825 weather stations across China to a spatial field with grid spacing of 500m. Then, the values of each grid located in one county were averaged. We only used county-level weather data for the counties in the five provinces. The units of measure are degrees centigrade for temperature, and millimeters for precipitation. The period we construct the weather variables over encompasses the early and late growing seasons in a year; specifically, this period is defined as Mar 15 - Oct 15 for counties in Fujian, Jiangxi, and Guangxi, and Mar 15 - Nov 15 for counties in Guangdong and Hainan.[1]

Following the literature on climate change impacts on crop yields[4,5], we construct three weather variables over the period defined above: (1) growing degree days, (2) heating degree days, and (3) total precipitation. We define growing degree days as follows. We denote the average temperature in county i in province r on day d in year t as $T_{irt,d}$, and define the daily degree days (8°C - 29°C) as

$$D_{irt,d} = \begin{cases} 0 & \text{if } T_{irt,d} < 8; \\ T_{irt,d} - 8 & \text{if } 8 \leq T_{irt,d} \leq 29; \\ 21 & \text{if } T_{irt,d} > 29, \end{cases} \tag{5}$$

After calculating $D_{irt,d}$ for each county i in province r for each day d in year t, we sum over the entire growing season and obtain the growing degree days GD_{irt} for each county i in year t, i.e.,

$$GD_{irt} = \textstyle\sum_{d \in \mathbf{D}'} D_{irt,d}, \tag{6}$$

where $\mathbf{D}'$ denotes all days in the period defined earlier. We define heating degree days as

$$HD_{irt} = \sum_{d \in \mathbf{D}'} \max\{T_{irt,d} - 29, 0\}. \tag{7}$$

[1]The sample period for the weather variables starts earlier than that for the ozone variables because heat and moisture matter for crops before their emergence, but ozone only starts to matter after crops have emerged.

Total precpitation TP_{irt} for each county i is defined as

$$TP_{irt} = \sum_{d \in \mathbf{D}'} P_{irt,d}, \tag{8}$$

where $P_{irt,d}$ represents the total precipitation in county i in day d of year t.

In the regression results, these environmental factors are divided by 1000 to scale up the magnitude of coefficients. This adjustment does not influence the estimation of other variables and the interpretation of their coefficients.

Fertilizer application

The fertilizer data provide a nutrient-based fertilizer index for each province. The index is calculated based on effective chemical compounds, namely nitrogen, phosphorus, and potassium, specifically applied on rice on a per-hectare basis. The measure is comparable across regions because it is based on the quantity of effective nutrients. In the raw data, the fertilizer index is provided separately for four different categories, namely early Indica rice, middle Indica rice, late Indica rice, and single-cropped Japonica rice. For each province in each year, the four measures are weighted by the share of production corresponding to each category, and a single index of fertilizer use is obtained for each province-year combination.[2]

Natural disasters

Four province-level statistics are reported for natural disasters: area covered by floods, area covered by droughts, area affected by floods, and area affected by droughts. The measure of *area covered* is defined as the area where the disaster reduces more than 10% of the total crop production; and *area affected* is defined as the area where the disaster reduces more than 30% of the total crop production. To make the measure comparable between different provinces, we calculate a disaster severity index using a ratio-based measure. In particular, we obtain the total arable area at the provincial level, and divide area covered and area affected by the total arable area for every province. We place these ratios into four categories: flood (severe or mild) and drought (severe or mild).

IV. Measuring nonlinearities in O_3 damage to rice yields

We construct daily maximum bins to capture the effects of extreme values of O_3. Each bin counts days with the daily maximum O_3 concentration falling into a pre-determined range. Specifically, bins are defined as

$$o_{irt,b1} = \sum_{d \in \mathbf{D}} \mathbf{1}\Big\{ \max \left\{ o_{irt,d,h} \right\}_{h \in [10,17]} \in [60, 80) \Big\} \tag{9}$$

$$o_{irt,b2} = \sum_{d \in \mathbf{D}} \mathbf{1}\Big\{ \max \left\{ o_{irt,d,h} \right\}_{h \in [10,17]} \in [80, 100) \Big\} \tag{10}$$

[2]The data compilation provides fertilizer data for major producing provinces, including Fujian, Guangdong, Guangxi, and Jiangxi. Data are not available for Hainan, and thus the fertilizer use for that small province is set to zero.

$$o_{irt,b3} = \sum_{d \in \mathbf{D}} \mathbf{1}\Big\{ \max \left\{ o_{irt,d,h} \right\}_{h \in [10,17]} \in [100, 120) \Big\} \tag{11}$$

$$o_{irt,b4} = \sum_{d \in \mathbf{D}} \mathbf{1}\Big\{ \max \left\{ o_{irt,d,h} \right\}_{h \in [10,17]} \geq 120 \Big\} \tag{12}$$

We construct these bins only during panicle formation, i.e., $\mathbf{D}$ in equations 9 - 12 corresponds to PF in Table 2. We use the same variables to control for environmental factors, fertilizer application, and natural disasters as in the baseline regression. The regression equation can be writen as

$$\log(y_{irt}) = \beta_1 o^{PF}_{irt,b1} + \beta_2 o^{PF}_{irt,b2} + \beta_3 o^{PF}_{irt,b3} + \beta_4 o^{PF}_{irt,b4} + \mathbf{w}'_{irt}\gamma + \mathbf{x}'_{rt}\delta + \alpha_{ir} + \lambda_t + \varepsilon_{irt}. \tag{13}$$

V. Discussion of the Results

Table 3 shows the estimated results for equation (1) using simple average O_3, SUM60, and AOT40. Among the coefficients associated with O_3 variables, only variables during PF are statistically significant. A one ppb increase in daytime average O_3 during PF is associated with a 0.48% decrease in rice yield. A one ppmh increase in SUM60 (AOT40) is associated with a 0.74% (1.56%) decrease in rice yield.

Table 4 presents estimated results for equation (13). Only coefficients associated with daily maximum O_3 above 100 ppb are statistically significant. Specifically, during PF, one more day with its maximum O_3 over 100 (120) ppb is associated with a 0.94% (1.28%) decrease in rice yield. This means the accumulation of extremely high O_3 exposures is particularly detrimental to rice yields.

The weather variables are not statistically significant. In contrast to statistical studies on climate change and crop yields using long panels, we use a short panel to estimate the relationship between yield and O_3 pollution. Because our short panel consists of only three years, the year-to-year variation in weather variables is not substantial and hence we do not find statistically significant weather effects on yields using our data set. Among other control variables, only the fertilizer index displays a significantly positive effect on rice yields.

Table 5 shows the decomposition of variation in rice yields explained by different sets of explanatory variables. We focus on the within-R^2 of a certain set of explanatory variables because the explanatory power in our estimation strategy is mostly provided by within-county variation. The ratio in parentheses below the within-R^2 indicates the portion of yield variation explained by the corresponding set of variables. It is evident that most of the within-county variation comes from O_3 variables. For the control variables, while fertilizer application and natural disasters provide moderate explanatory power, only a very small portion of yield variation is explained by the variation in weather variables.

To address any concern of potential multi-collinearity, we perform correlation tests conditional on the fixed effects. It is worth noting that the identification of partial effects in our estimation strategy relies on year-by-year variation within the same county. Hence, the multi-collinearity issue arises only if the within-county variation of different explanatory variables are highly correlated. We show results of the correlation tests in Tables 6 and 7. The former controls for county fixed effects, and the latter controls for both county and year fixed effects. We do not find high correlation between O_3 variables and other explanatory variables and therefore we are confident that our estimation of O_3 coefficients are not subject to a multi-collinearity problem.

To verify that our empirical results are not sensitive to the chosen daytime window, we perform sensitivity analyses by varying the definition of daytime windows. We repeat our analysis by using the following time windows: 9am-5pm, 10am-6pm, 9am-6pm, 8am-6pm, 9am-7pm, and 8am-7pm. The estimated coefficients associated with O_3 variables are reported in Table 8. The results are consistent with our findings as in Tables 3 and 4.

We estimate the yield changes from 2006 to 2010 caused by variation in O_3 during PF based on our regression results. Figure 3 shows the spatial distribution of the impact based on the three O_3 measures: simple average, SUM60, and AOT40. The three different measures produce consistent estimates of the impact from 2006 to 2010. While some counties in Guangxi, Fujian, and Jiangxi experienced some yield recovery due to the alleviation in O_3 during PF, the majority of counties, especially in Guangdong, experienced substantial yield losses because of the higher O_3 pollution during PF in 2010 relative to 2006.

VI. O_3 damage over the entire growing season

We use an alternative model to study the O_3 damage ignoring differences across stages of plant growth. We construct the 8-hour simple average, SUM60, and AOT40 using the entire growing season (GS). These regression results are presented in Table 9. We find that a 1 ppb increase in GS simple average concentration lowers yield by 0.60% ±0.34%, whereas an additional ppmh in GS SUM60 and AOT40 is associated with a 0.16% ±0.16% and 0.42% ±0.31% yield loss, respectively.

Figure 2 compares the coefficients estimated from models with and without the stage specificity. The GS coefficient of the simple average measure is similar to the PF coefficient in the baseline, while the SUM60 and AOT40 coefficients are smaller in magnitude than their PF counterparts. This is because SUM60 and AOT40 are not "average measures". A one ppb decrease in simple average ozone concentration for GS implies a one ppb decrease for PF, which is consistent with the finding that GS and PF coefficients are very close. However, a one ppmh reduction in SUM60 or AOT40 over the entire GS is equivalent to a reduction of 1/3 ppmh before, during and after PF. Since changes in SUM60 or AOT40 before and after PF are not associated with yield changes at a statistically significant level, the 1 ppmh reduction in SUM60 or AOT40 over the entire GS has a similar impact as a 1/3 ppmh reduction during PF. Therefore the benefit arising from this reduction would be roughly a third of a 1ppmh reduction during PF. To confirm our reasoning, using our regression estimates for PF, a 1/3 ppmh reduction in SUM60 and AOT40 would reduce yields by 0.25% ±0.17% and 0.53% ±0.38%, respectively, which are similar up to sampling variability to the estimates of a 1 ppmh reduction in SUM60 and AOT40 over the GS.

We believe that our GS estimates are underestimates of O_3 damage over the entire growing season because the potential omitted variables drive point estimates of pre- and post-PF effects to be positive (although statistically insignificant), while positive coefficients are illogical since no evidence shows O_3 can potentially benefit crop growth. An experimental study in China[6] concludes that one additional ppmh of AOT40 is associated with a 0.95% reduction in rice yield, which is larger in magnitude than our GS estimate of a 1 ppmh increase in AOT40. It is important to note that these two estimates are not readily comparable as our definition of GS encompasses two consecutive growing seasons, we only observe average yield for both seasons and our sample is not localized to a small region as in an experimental study. If we assume an additional ppmh is evenly increased across two seasons and its impact is homogeneous across the two seasons, the

yield reduction associated with a 1 ppmh increase in AOT40 will be roughly 0.84% based on our GS estimate, which is slightly lower than 0.95%.

Tang et al. (2014)[7] estimate the increases in ozone risk in terms of AOT40 from 2000 to 2020 for different regions of China, and calculate the loss of rice harvest by using the yield-ozone relationship established in Wang et al. (2013)[6]. Tang et al. (2014) project that rice production loss will be as high as 7.9 - 12.1% from 2000 to 2020 due to ozone pollution. If this projection was based on our GS estimate using AOT40, the projected production loss would be around 7.0 - 10.7%. Hence, our results using county-level longitudinal data confirm the findings in Tang et al (2014).

References

[1] Hiroko Sawada and Yoshihisa Kohno. Differential ozone sensitivity of rice cultivars as indicated by visible injury and grain yield. *Plant Biology*, 11(1), 2009.

[2] Yunxia Wang, Lianxin Yang, Kazuhiko Kobayashi, Jianguo Zhu, Charles P Chen, Kaifang Yang, Haoye Tang, and Yulong Wang. Investigations on spikelet formation in hybrid rice as affected by elevated tropospheric ozone concentration in china. *Agriculture, Ecosystems & Environment*, 150:63–71, 2012.

[3] Keita Tsukahara, Hiroko Sawada, Yoshihisa Kohno, Takakazu Matsuura, Izumi Mori, Tomio Terao, Motohide Ioki, and Masanori Tamaoki. Ozone-induced rice grain yield loss is triggered via a change in panicle morphology that is controlled by aberrant panicle organization 1 gene. *PLOS ONE*, 10(4), 2015.

[4] Wolfram Schlenker, W Michael Hanemann, and Anthony C Fisher. The impact of global warming on us agriculture: an econometric analysis of optimal growing conditions. *Review of Economics and Statistics*, 88(1):113–125, 2006.

[5] Wolfram Schlenker and Michael J Roberts. Nonlinear temperature effects indicate severe damages to us crop yields under climate change. *Proceedings of the National Academy of Sciences*, 106(37):15594–15598, 2009.

[6] Yuxuan Wang, Lulu Shen, Shiliang Wu, Loretta Mickley, Jingwei He, and Jiming Hao. Sensitivity of surface ozone over China to 2000-2050 global changes of climate and emissions. *Atmospheric Environment*, 75:374–382, 2013.

[7] Haoye Tang, Jing Pang, Gongxuan Zhang, Masayuki Takigawa, Gang Liu, Jianguo Zhu, and Kazuhiko Kobayashi. Mapping ozone risks for rice in china for years 2000 and 2020 with flux-based and exposure-based doses. *Atmospheric Environment*, 86:74–83, 2014.

Figure 1: Trends in China's Rice Production and Trade.

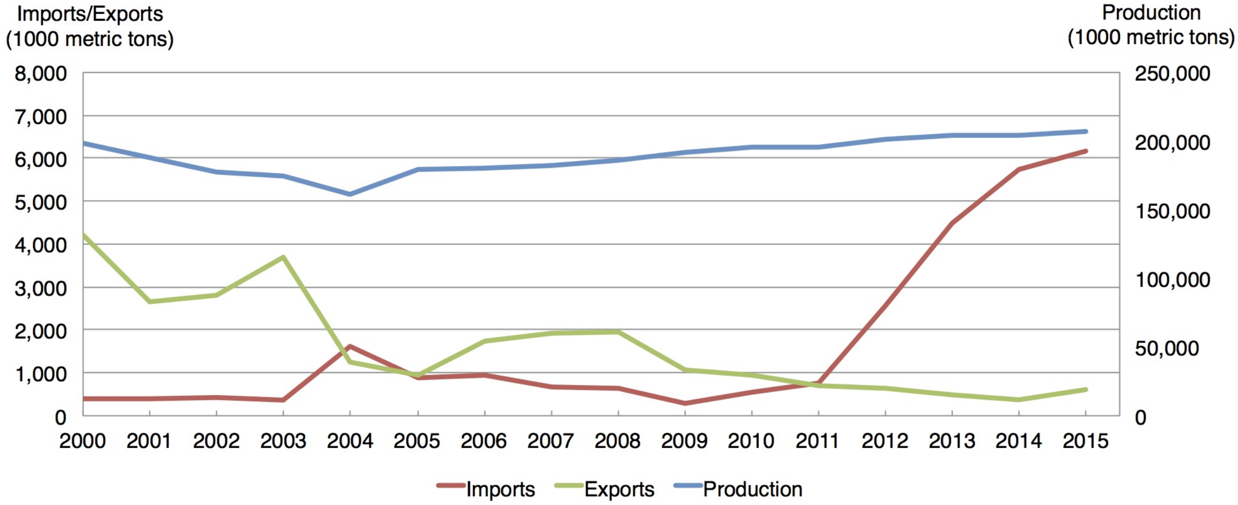

Note: All data are on a rough rice (i.e., paddy) basis and are obtained from the PSD online database at the USDA Foreign Agriculture Service (http://apps.fas.usda.gov/psdonline/).

Figure 2: Comparing Coefficients With and Without Stage Specificity.

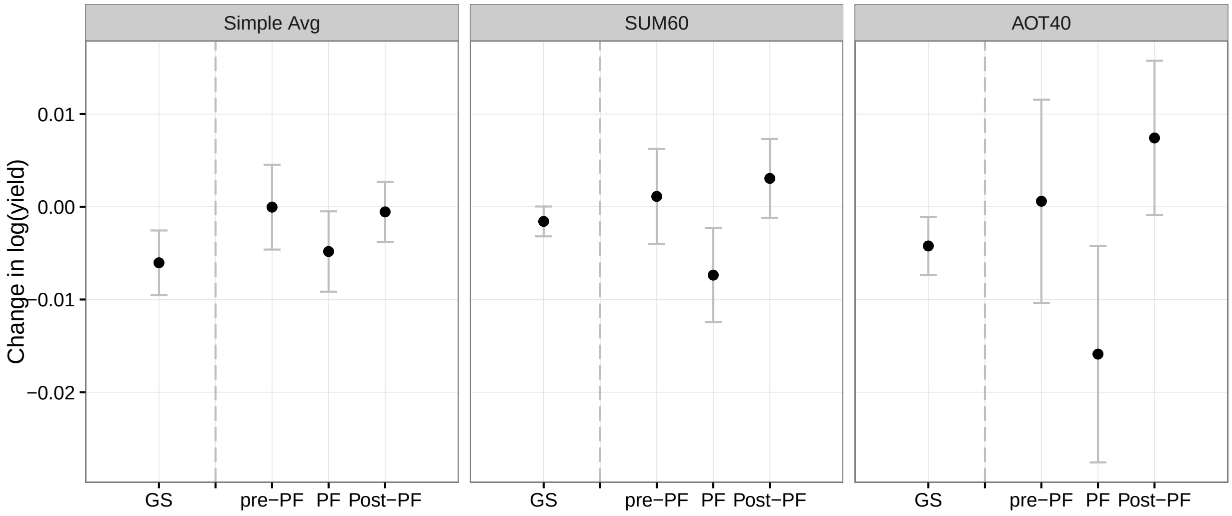

Note: This figure compares ozone coefficients obtained from a regression using the simple average, SUM60 and AOT40 constructed over the entire GS with those constructed separately over three periods: the period before PF (Pre-PF), the PF period (PF), and the period after PF (Post-PF). Black dots represent point estimates, and gray bars represent 95% confidence intervals based on prefecture-level cluster-robust standard errors. The regression includes control variables for weather, fertilizer application, and natural disasters, as well as county and year fixed effects.

Figure 3: Estimated Yield Changes Associated with O_3 Variation: 2006-2010.

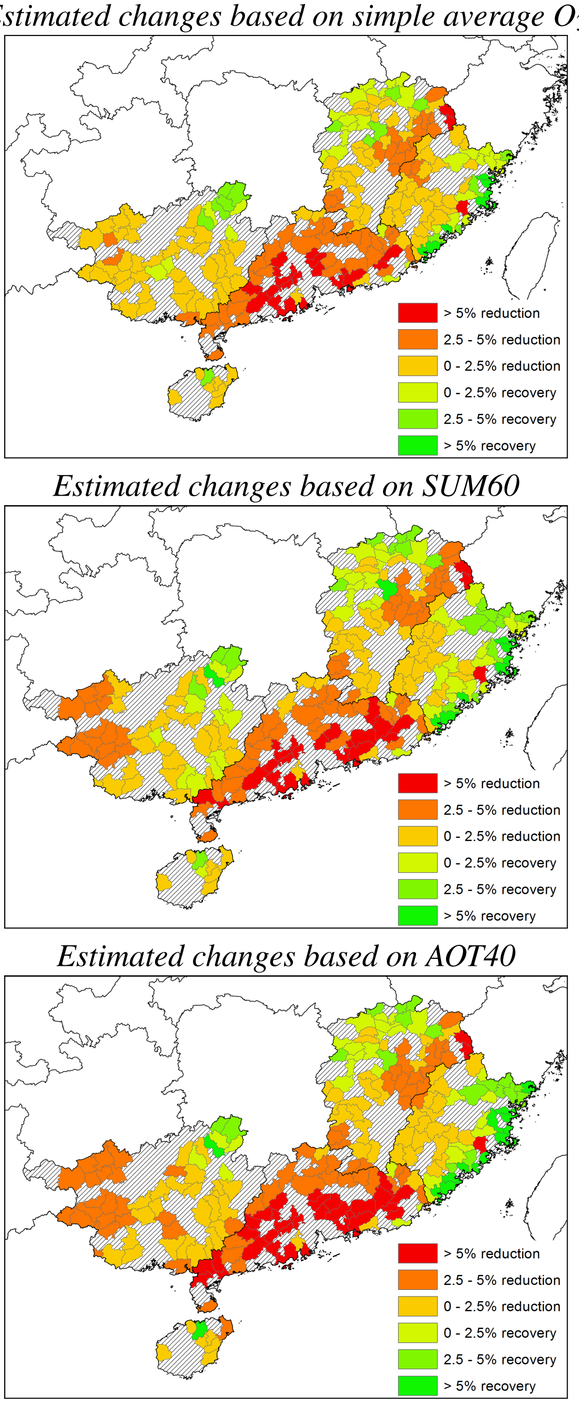

Note: Magnitudes of changes are estimated by multiplying PF coefficients reported in Table 3 and within-county variation from 2006 to 2010 of the corresponding O_3 variables . The within-county variation is calculated by subtracting the value of PF O_3 variable in 2010 from its counterpart in 2006. These maps were generated by mapping estimated values from our data to county polygons by using ArcGIS 10 (http://www.esri.com/software/arcgis). The shapefile of polygons was obtained from Global Administrative Areas (http://gadm.org).

Figure 4: Levels and Variations of SUM60 during PF over 2006 – 2010.

SUM60 during PF averaged across 2006, 2008, and 2010

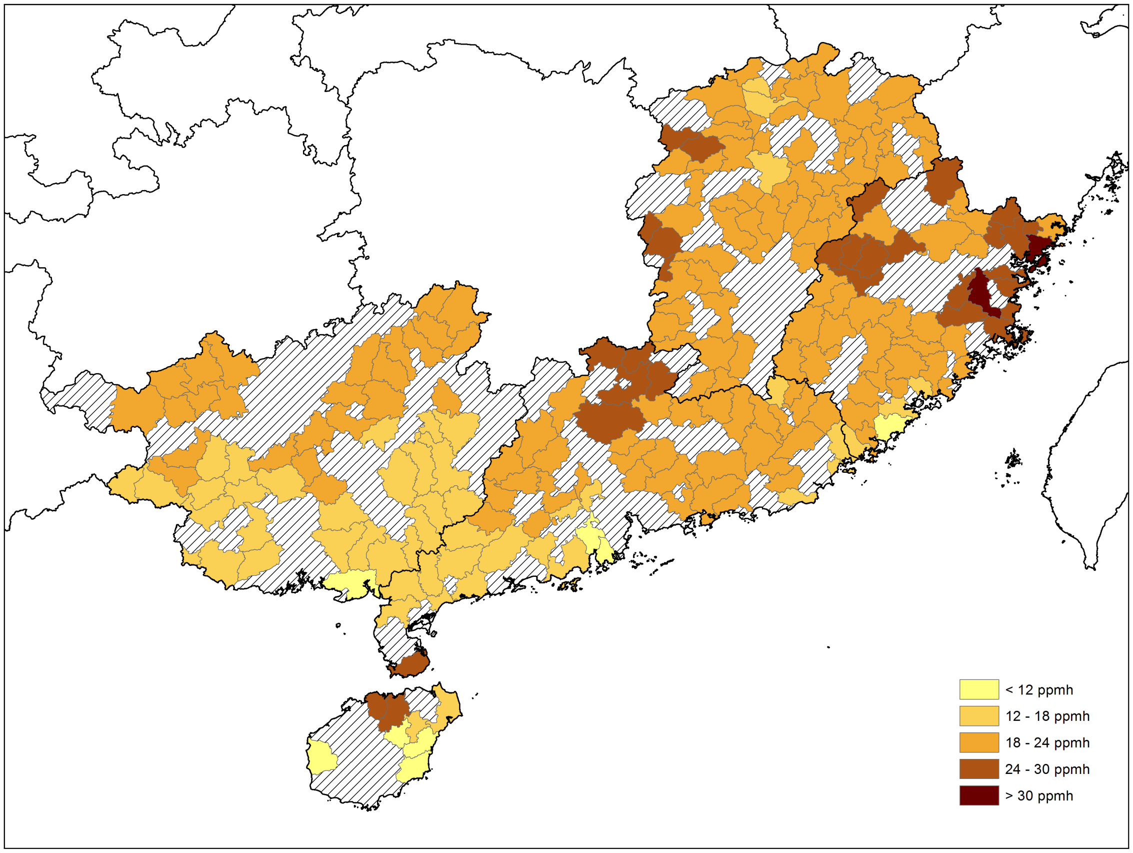

Year-to-year variation of SUM60 during PF

Changes from 2006 to 2008

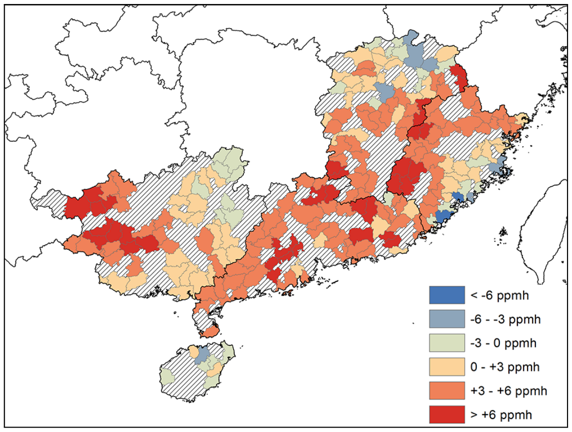

Changes from 2008 to 2010

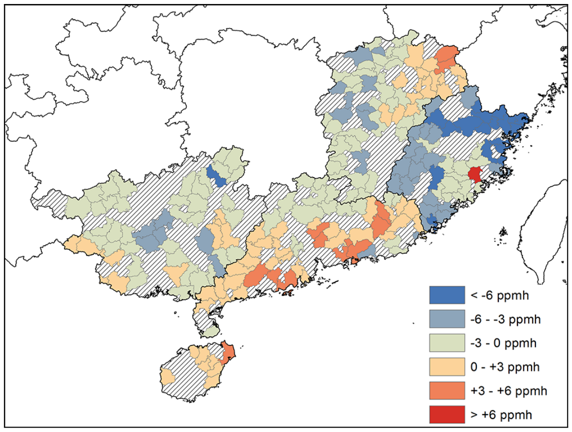

Note: Counties shown in color are used in the regression analysis. Black and white shaded areas are not included because they did not plant rice consistently over the time period analyzed. Regions shown in white are outside the five provinces in our sample. These maps were generated by mapping calculated values from our data to county polygons by using ArcGIS 10 (http://www.esri.com/software/arcgis). The shapefile of polygons was obtained from Global Administrative Areas (http://gadm.org).

Figure 5: Levels and Variations of AOT40 during PF over 2006 – 2010.

AOT40 during PF averaged across 2006, 2008, and 2010

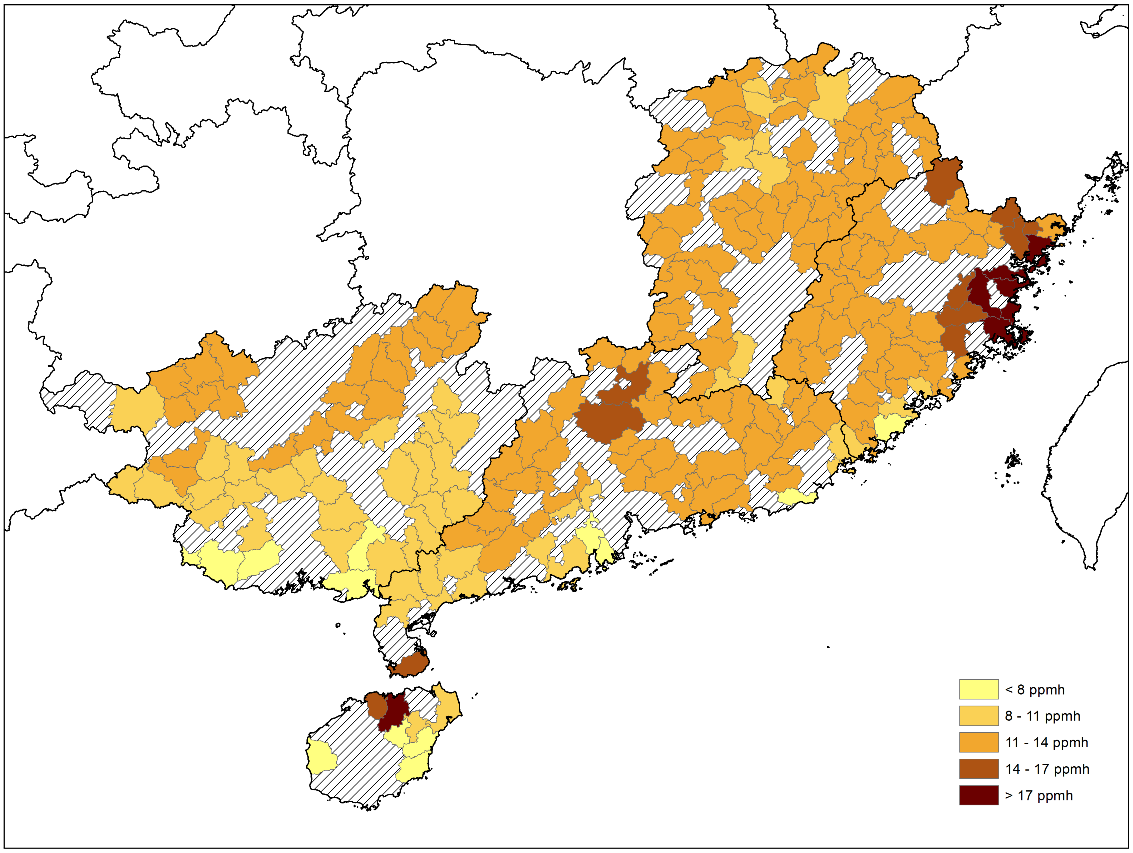

Year-to-year variation of AOT40 during PF

Changes from 2006 to 2008

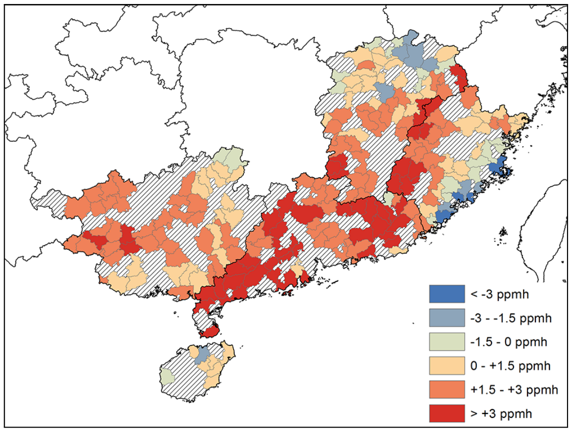

Changes from 2008 to 2010

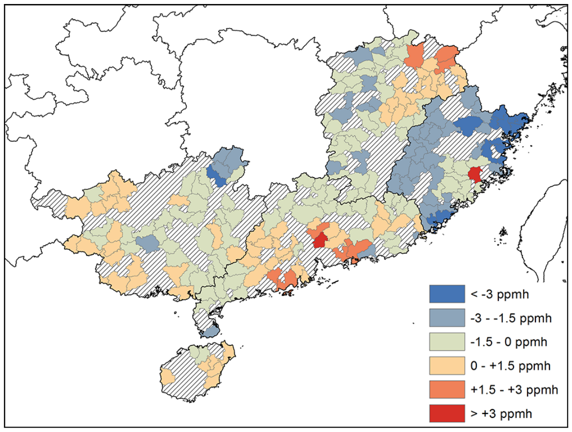

Note: Counties shown in color are used in the regression analysis. Black and white shaded areas are not included because they did not plant rice consistently over the time period analyzed. Regions shown in white are outside the five provinces in our sample. These maps were generated by mapping calculated values from our data to county polygons by using ArcGIS 10 (http://www.esri.com/software/arcgis). The shapefile of polygons was obtained from Global Administrative Areas (http://gadm.org).

Table 1: Total Rice Production and Planted Area for the Sample and the Entire Country.

**Year**	**Rice Output** (mmt)			**Rice Planted Area** (mha)		
	Country	Sample	Ratio	Country	Sample	Ratio
2006	181.72	34.69	19.09%	28.94	5.91	20.42%
2008	191.90	32.96	17.18%	29.24	5.83	19.94%
2010	195.76	33.72	17.23%	29.87	5.88	19.69%

Note: Values for the entire country are obtained from National Bureau of Statistics of China. Values for the sample are calculated based on observations used for the regression. All data are on a rough rice (i.e., paddy) basis.

Table 2: Dates of Growing Stages.

Province	Early growing season			Late growing season		
	pre-PF	PF	post-PF	pre-PF	PF	post-PF
Fujian, Jiangxi, Guangxi	4/16 - 5/15	5/16 - 6/15	6/16 - 7/15	7/16 - 8/15	8/16 - 9/15	9/16 - 10/15
Guangdong, Hainan	4/16 - 5/15	5/16 - 6/15	6/16 - 7/15	8/1 - 8/31	9/1 - 9/30	10/1 - 11/15

Note: Dates are determined according to the rice progress report by the National Bureau of Statistics of China.

Table 3: Regression Results Using Simple Average, SUM60, AOT40 over Different Stages.

	Simple Avg.	SUM60	AOT40
O_3: pre-PF	0.0000	0.0011	0.0006
	(0.00228)	(0.00255)	(0.00546)
O_3: PF	-0.0048	-0.0074	-0.0159
	(0.00216)**	(0.00253)***	(0.00582)***
O_3: post-PF	-0.0005	0.0031	0.0074
	(0.00161)	(0.00212)	(0.00415)*
Growing degree days (1000°C)	0.0422	0.0411	0.0335
	(0.07633)	(0.07459)	(0.07365)
Heating degree days (1000°C)	0.0503	0.1143	0.2133
	(0.45073)	(0.45267)	(0.44052)
Total precipitation (1000mm)	-0.0049	-0.0064	-0.0028
	(0.01747)	(0.01981)	(0.01771)
Fertilizer index (kg/ha)	0.0028	0.0029	0.0030
	(0.00136)**	(0.00116)**	(0.00120)**
Flood coverage (severe)	0.5191	0.4688	0.5573
	(0.38354)	(0.34047)	(0.36342)
Drought coverage (severe)	1.5383	1.6339	1.5202
	(0.92781)	(0.99213)	(0.86025)*
Flood coverage (mild)	0.4423	0.5531	0.5870
	(0.27907)	(0.28045)*	(0.29600)*
Drought coverage (mild)	-0.3797	-0.2787	-0.1397
	(0.72999)	(0.72656)	(0.66570)
County & year fixed effects	Yes	Yes	Yes
Observations	693	693	693
Overall R^2	0.858	0.856	0.861
Within R^2	0.283	0.271	0.296

Note: This table reports the regression results using ozone variables constructed separately before, during, and after panicle formation (pre-PF, PF, and post-PF). The ozone variable used in the regression is indicated by the column header. Prefecture-level cluster-robust standard errors are reported in parentheses.

Significance levels: * $p < 0.10$, ** $p < 0.05$, *** $p < 0.01$

Table 4: Regression Using the Number of Days in Different Categories Based on the 8-hour Maximum Ozone Concentration.

	Coef.
Daily Maximum: 60 - 80 ppb	-0.0010
	(0.00107)
Daily Maximum: 80 - 100 ppb	0.0007
	(0.00123)
Daily Maximum: 100 - 120 ppb	-0.0094
	(0.00220)***
Daily Maximum: $>$ 120 ppb	-0.0128
	(0.00440)***
Growing degree days (1000°C)	0.0009
	(0.07016)
Heating degree days (1000°C)	0.2924
	(0.42799)
Total precipitation (1000mm)	0.0077
	(0.01602)
Fertilizer index (kg/ha)	0.0035
	(0.00114)***
Flood coverage (severe)	0.7927
	(0.34972)**
Drought coverage (severe)	1.0158
	(0.87769)
Flood coverage (mild)	0.5762
	(0.25791)**
Drought coverage (mild)	-0.0169
	(0.64000)
County & Year FE	Yes
Observations	693
Overall R^2	0.868
Within R^2	0.334

Note: O_3 variables are constructed over PF. Each O_3 bin counts the number of days with the maximum daytime O_3 falling in that bin. Coefficients are estimated based on regression equation (13). Prefecture-level cluster-robust standard errors are reported in parentheses.
Significance levels: * $p < 0.10$, ** $p < 0.05$, *** $p < 0.01$

Table 5: Yield Variation Explained by Various Sets of Explanatory Variables.

Variables Included	Simple Avg.		SUM60		AOT40		O_3 Bins	
	Within-R^2	R^2	Within-R^2	R^2	Within-R^2	R^2	Within-R^2	R^2
(1): O_3 + FE's	0.2051	0.8422	0.1598	0.8332	0.1810	0.8374	0.1792	0.8371
	(72.50%)		(58.15%)		(60.45%)		(53.65%)	
(2): (1) + Weather	0.2062	0.8424	0.1615	0.8335	0.1822	0.8376	0.1799	0.8372
	(72.89%)		(58.77%)		(60.86%)		(53.86%)	
(3): (2) + Fertilizer	0.2161	0.8444	0.1852	0.8382	0.2034	0.8419	0.2180	0.8448
	(76.39%)		(67.39%)		(67.94%)		(65.27%)	
(4): (3) + Disasters	0.2829	0.8577	0.2748	0.8560	0.2994	0.8609	0.3340	0.8678
	(100.00%)		(100.00%)		(100.00%)		(100.00%)	

Note: The R^2 statistics are obtained by regressing the dependent variable on a certain set of explanatory variables (indicated in "Variables Included"). The ratio in the parentheses indicates the portion of yield variation explained by the corresponding set of explanatory variables. Fixed effects (FE's) include both county and year fixed effects.

Table 6: Correlation Matrix of Explanatory Variables Conditional on County Fixed Effects.

**Simple Avg. Regression**	(1)	(2)	(3)	(4)	(5)	(6)	(7)	(8)	(9)	(10)	(11)
(1) O_3: pre-PF	1.00										
(2) O_3: PF	0.33	1.00									
(3) O_3: post-PF	0.29	0.34	1.00								
(4) Growing degree days	0.02	0.02	-0.05	1.00							
(5) Heating degree days	0.01	0.01	-0.01	0.48	1.00						
(6) Total precipitation	0.02	0.01	0.05	-0.28	-0.20	1.00					
(7) Fertilizer index	-0.10	0.20	-0.31	0.05	-0.12	-0.42	1.00				
(8) Flood coverage (severe)	-0.06	0.03	0.19	-0.36	-0.14	0.49	-0.69	1.00			
(9) Drought coverage (severe)	-0.09	0.08	-0.39	0.02	0.03	0.02	0.27	-0.02	1.00		
(10) Flood coverage (mild)	-0.46	0.04	-0.13	0.07	0.12	0.04	0.09	0.09	0.17	1.00	
(11) Drought coverage (mild)	0.06	0.19	-0.23	-0.18	-0.16	0.02	0.48	-0.01	0.63	0.12	1.00

**SUM60 Regression**	(1)	(2)	(3)	(4)	(5)	(6)	(7)	(8)	(9)	(10)	(11)
(1) O_3: pre-PF	1.00										
(2) O_3: PF	0.32	1.00									
(3) O_3: post-PF	0.20	0.22	1.00								
(4) Growing degree days	-0.02	0.01	-0.12	1.00							
(5) Heating degree days	-0.03	0.03	-0.08	0.48	1.00						
(6) Total precipitation	0.02	-0.01	0.10	-0.28	-0.20	1.00					
(7) Fertilizer index	-0.06	0.15	-0.38	0.05	-0.12	-0.42	1.00				
(8) Flood coverage (severe)	-0.08	0.06	0.31	-0.36	-0.14	0.49	-0.69	1.00			
(9) Drought coverage (severe)	-0.13	0.04	-0.30	0.02	0.03	0.02	0.27	-0.02	1.00		
(10) Flood coverage (mild)	-0.45	0.07	-0.15	0.07	0.12	0.04	0.09	0.09	0.17	1.00	
(11) Drought coverage (mild)	0.03	0.19	-0.31	-0.18	-0.16	0.02	0.48	-0.01	0.63	0.12	1.00

**AOT40 Regression**	(1)	(2)	(3)	(4)	(5)	(6)	(7)	(8)	(9)	(10)	(11)
(1) O_3: pre-PF	1.00										
(2) O_3: PF	0.27	1.00									
(3) O_3: post-PF	0.23	0.27	1.00								
(4) Growing degree days	-0.01	-0.02	-0.04	1.00							
(5) Heating degree days	0.00	0.05	-0.05	0.48	1.00						
(6) Total precipitation	0.00	0.07	0.09	-0.28	-0.20	1.00					
(7) Fertilizer index	-0.09	0.09	-0.35	0.05	-0.12	-0.42	1.00				
(8) Flood coverage (severe)	-0.06	0.14	0.22	-0.36	-0.14	0.49	-0.69	1.00			
(9) Drought coverage (severe)	-0.14	0.06	-0.30	0.02	0.03	0.02	0.27	-0.02	1.00		
(10) Flood coverage (mild)	-0.44	0.15	-0.15	0.07	0.12	0.04	0.09	0.09	0.17	1.00	
(11) Drought coverage (mild)	0.02	0.21	-0.30	-0.18	-0.16	0.02	0.48	-0.01	0.63	0.12	1.00

**Daily Max. Regression**	(1)	(2)	(3)	(4)	(5)	(6)	(7)	(8)	(9)	(10)	(11)	(12)
(1) Daily Max.: 60 - 80 ppb	1.00											
(2) Daily Max.: 80 - 100 ppb	-0.05	1.00										
(3) Daily Max.: 100 - 120 ppb	-0.07	-0.13	1.00									
(4) Daily Max.: $>$120 ppb	-0.22	-0.17	-0.06	1.00								
(5) Growing degree days	-0.01	0.10	-0.04	-0.08	1.00							
(6) Heating degree days	0.01	0.05	-0.01	-0.06	-0.06	1.00						
(7) Total precipitation	-0.09	-0.18	0.01	0.21	-0.30	-0.09	1.00					
(8) Fertilizer index	0.16	-0.02	0.01	-0.06	-0.62	0.43	-0.02	1.00				
(9) Flood coverage (severe)	-0.18	-0.08	0.09	0.27	0.15	-0.37	0.32	-0.72	1.00			
(10) Drought coverage (severe)	0.04	0.03	-0.02	-0.01	-0.52	0.41	0.13	0.77	-0.41	1.00		
(11) Flood coverage (mild)	-0.30	0.12	0.09	0.23	0.38	-0.16	-0.04	-0.41	0.32	-0.27	1.00	
(12) Drought coverage (mild)	-0.08	-0.16	0.07	0.10	-0.58	0.29	0.14	0.79	-0.40	0.83	-0.27	1.00

Note: Each panel reports the correlation matrix of explanatory variables used in a specific regression. The regression is indicated at the top-left of the panel. To obtain conditional correlations, each variable is first regressed on the fixed effects, and the residual is used for calculating correlations.

Table 7: Correlation Matrix of Explanatory Variables Conditional on County and Year Fixed Effects.

**Simple Avg. Regression**	(1)	(2)	(3)	(4)	(5)	(6)	(7)	(8)	(9)	(10)	(11)
(1) O_3: pre-PF	1.00										
(2) O_3: PF	0.33	1.00									
(3) O_3: post-PF	0.29	0.34	1.00								
(4) Growing degree days	0.02	0.02	-0.05	1.00							
(5) Heating degree days	0.01	0.01	-0.01	0.48	1.00						
(6) Total precipitation	0.02	0.01	0.05	-0.28	-0.20	1.00					
(7) Fertilizer index	-0.10	0.20	-0.31	0.05	-0.12	-0.42	1.00				
(8) Flood coverage (severe)	-0.06	0.03	0.19	-0.36	-0.14	0.49	-0.69	1.00			
(9) Drought coverage (severe)	-0.09	0.08	-0.39	0.02	0.03	0.02	0.27	-0.02	1.00		
(10) Flood coverage (mild)	-0.46	0.04	-0.13	0.07	0.12	0.04	0.09	0.09	0.17	1.00	
(11) Drought coverage (mild)	0.06	0.19	-0.23	-0.18	-0.16	0.02	0.48	-0.01	0.63	0.12	1.00

**SUM60 Regression**	(1)	(2)	(3)	(4)	(5)	(6)	(7)	(8)	(9)	(10)	(11)
(1) O_3: pre-PF	1.00										
(2) O_3: PF	0.32	1.00									
(3) O_3: post-PF	0.20	0.22	1.00								
(4) Growing degree days	-0.02	0.01	-0.12	1.00							
(5) Heating degree days	-0.03	0.03	-0.08	0.48	1.00						
(6) Total precipitation	0.02	-0.01	0.10	-0.28	-0.20	1.00					
(7) Fertilizer index	-0.06	0.15	-0.38	0.05	-0.12	-0.42	1.00				
(8) Flood coverage (severe)	-0.08	0.06	0.31	-0.36	-0.14	0.49	-0.69	1.00			
(9) Drought coverage (severe)	-0.13	0.04	-0.30	0.02	0.03	0.02	0.27	-0.02	1.00		
(10) Flood coverage (mild)	-0.45	0.07	-0.15	0.07	0.12	0.04	0.09	0.09	0.17	1.00	
(11) Drought coverage (mild)	0.03	0.19	-0.31	-0.18	-0.16	0.02	0.48	-0.01	0.63	0.12	1.00

**AOT40 Regression**	(1)	(2)	(3)	(4)	(5)	(6)	(7)	(8)	(9)	(10)	(11)
(1) O_3: pre-PF	1.00										
(2) O_3: PF	0.27	1.00									
(3) O_3: post-PF	0.23	0.27	1.00								
(4) Growing degree days	-0.01	-0.02	-0.04	1.00							
(5) Heating degree days	0.00	0.05	-0.05	0.48	1.00						
(6) Total precipitation	0.00	0.07	0.09	-0.28	-0.20	1.00					
(7) Fertilizer index	-0.09	0.09	-0.35	0.05	-0.12	-0.42	1.00				
(8) Flood coverage (severe)	-0.06	0.14	0.22	-0.36	-0.14	0.49	-0.69	1.00			
(9) Drought coverage (severe)	-0.14	0.06	-0.30	0.02	0.03	0.02	0.27	-0.02	1.00		
(10) Flood coverage (mild)	-0.44	0.15	-0.15	0.07	0.12	0.04	0.09	0.09	0.17	1.00	
(11) Drought coverage (mild)	0.02	0.21	-0.30	-0.18	-0.16	0.02	0.48	-0.01	0.63	0.12	1.00

**Daily Max. Regression**	(1)	(2)	(3)	(4)	(5)	(6)	(7)	(8)	(9)	(10)	(11)	(12)
(1) Daily Max.: 60 - 80 ppb	1.00											
(2) Daily Max.: 80 - 100 ppb	-0.34	1.00										
(3) Daily Max.: 100 - 120 ppb	-0.12	-0.20	1.00									
(4) Daily Max.: >120 ppb	-0.27	-0.23	-0.07	1.00								
(5) Growing degree days	0.13	0.16	-0.07	-0.18	1.00							
(6) Heating degree days	-0.03	0.11	0.00	-0.02	0.48	1.00						
(7) Total precipitation	-0.09	-0.17	0.02	0.23	-0.28	-0.20	1.00					
(8) Fertilizer index	0.15	-0.04	0.07	0.02	0.05	-0.12	-0.42	1.00				
(9) Flood coverage (severe)	-0.17	-0.13	0.09	0.28	-0.36	-0.14	0.49	-0.69	1.00			
(10) Drought coverage (severe)	-0.05	0.09	-0.01	0.07	0.02	0.03	0.02	0.27	-0.02	1.00		
(11) Flood coverage (mild)	-0.29	0.18	0.09	0.23	0.07	0.12	0.04	0.09	0.09	0.17	1.00	
(12) Drought coverage (mild)	-0.13	-0.10	0.16	0.25	-0.18	-0.16	0.02	0.48	-0.01	0.63	0.12	1.00

Note: Each panel reports the correlation matrix of explanatory variables used in a specific regression. The regression is indicated at the top-left of the panel. To obtain conditional correlations, each variable is first regressed on the fixed effects, and the residual is used for calculating correlations.

Table 8: Coefficients Obtained Using Alternative Daytime Windows.

	9am - 5pm	10am - 6pm	9am - 6pm	8am - 6pm	9am - 7pm	8am - 7pm
**Simple Avg. O_3**						
O_3: pre-PF	0.0002	0.0000	0.0003	0.0005	0.0004	0.0007
	(0.00243)	(0.00224)	(0.00238)	(0.00250)	(0.00235)	(0.00246)
O_3: PF	-0.0051	-0.0049	-0.0051	-0.0053	-0.0052	-0.0053
	(0.00233)**	(0.00218)**	(0.00233)**	(0.00244)**	(0.00236)**	(0.00246)**
O_3: post-PF	-0.0006	-0.0001	-0.0002	-0.0003	0.0001	0.0000
	(0.00176)	(0.00163)	(0.00177)	(0.00190)	(0.00181)	(0.00193)
**SUM60**						
O_3: pre-PF	0.0013	0.0012	0.0013	0.0015	0.0012	0.0013
	(0.00249)	(0.00217)	(0.00213)	(0.00210)	(0.00195)	(0.00193)
O_3: PF	-0.0072	-0.0069	-0.0067	-0.0065	-0.0064	-0.0062
	(0.00249)***	(0.00224)***	(0.00221)***	(0.00223)***	(0.00208)***	(0.00210)***
O_3: post-PF	0.0027	0.0032	0.0029	0.0027	0.0030	0.0028
	(0.00203)	(0.00186)*	(0.00179)	(0.00181)	(0.00164)*	(0.00166)
**AOT40**						
O_3: pre-PF	0.0010	0.0005	0.0009	0.0011	0.0009	0.0011
	(0.00532)	(0.00474)	(0.00464)	(0.00456)	(0.00421)	(0.00414)
O_3: PF	-0.0154	-0.0142	-0.0138	-0.0133	-0.0129	-0.0124
	(0.00565)***	(0.00514)***	(0.00500)***	(0.00491)***	(0.00462)***	(0.00454)***
O_3: post-PF	0.0072	0.0071	0.0069	0.0066	0.0068	0.0065
	(0.00405)*	(0.00369)*	(0.00362)*	(0.00358)*	(0.00334)**	(0.00332)*
**Daily Max. Hours**						
Daily Max.: 60 - 80 ppb	-0.0009	-0.0009	-0.0008	-0.0006	-0.0007	-0.0006
	(0.00095)	(0.00096)	(0.00086)	(0.00079)	(0.00077)	(0.00071)
Daily Max.: 80 - 100 ppb	0.0005	0.0005	0.0004	0.0006	0.0005	0.0005
	(0.00112)	(0.00110)	(0.00100)	(0.00091)	(0.00091)	(0.00083)
Daily Max.: 100 - 120 ppb	-0.0084	-0.0084	-0.0076	-0.0068	-0.0068	-0.0062
	(0.00201)***	(0.00194)***	(0.00180)***	(0.00163)***	(0.00164)***	(0.00150)***
Daily Max.: $>$120 ppb	-0.0114	-0.0115	-0.0103	-0.0092	-0.0094	-0.0085
	(0.00397)***	(0.00395)***	(0.00360)***	(0.00326)***	(0.00329)***	(0.00300)***

Note: The first three panels correspond to the specification using simple average, SUM60, and AOT40 with three stages. The last panel corresponds to the specification based on ozone bins during PF. In each panel, each column reports ozone coefficients with a different daytime window as indicated by the header. All regressions include control variables of weather, fertilizer application, and natural disasters. Prefecture-level cluster-robust standard errors are reported in parentheses.

Significance levels: * $p < 0.10$, ** $p < 0.05$, *** $p < 0.01$

Table 9: Regression Results Using Simple Average, SUM60, and AOT40 over the Entire Growing Season.

	Simple Avg.	SUM60	AOT40
O_3: GS	-0.0060	-0.0016	-0.0042
	(0.00174)***	(0.00080)*	(0.00156)***
Growing degree days (1000°C)	0.0290	0.0154	0.0195
	(0.07970)	(0.07981)	(0.07922)
Heating degree days (1000°C)	-0.0172	-0.0613	-0.0271
	(0.47808)	(0.49958)	(0.48372)
Total precipitation (1000mm)	-0.0083	-0.0101	-0.0091
	(0.01782)	(0.01872)	(0.01825)
Fertilizer index (kg/ha)	0.0013	0.0015	0.0013
	(0.00110)	(0.00115)	(0.00112)
Flood coverage (severe)	0.2671	0.3000	0.2913
	(0.31701)	(0.32106)	(0.31759)
Drought coverage (severe)	1.3627	1.5553	1.4310
	(0.94835)	(1.03370)	(0.98056)
Flood coverage (mild)	0.3363	0.4034	0.3857
	(0.23872)	(0.24815)	(0.24264)
Drought coverage (mild)	-0.1662	-0.3550	-0.2411
	(0.78367)	(0.85961)	(0.81426)
County & Year FE	Yes	Yes	Yes
Observations	693	693	693
Overall R^2	0.854	0.849	0.851
Within R^2	0.265	0.239	0.252

Note: This table reports the regression results using Simple average, SUM60, and AOT40 constructed over the entire growing season (GS). The ozone variable used in the regression is indicated by the column header. Prefecture-level cluster-robust standard errors are reported in the parentheses. Significance levels: * $p < 0.10$, ** $p < 0.05$, *** $p < 0.01$

Table 10: Year-to-Year Variation of Various Ozone Variables.

Simple Avg.		SUM60		AOT40	
Variation (ppb)	Proportion (%)	Variation (ppmh)	Proportion (%)	Variation (ppmh)	Proportion (%)
< -8	4.11	< -6	4.76	< -3	4.33
-8 - -4	10.17	-6 - -3	9.52	-3 - -1.5	11.04
-4 - 0	27.06	-3 - 0	28.79	- 1.5 - 0	27.06
0 - +4	25.32	0 - +3	25.11	0 - +1.5	25.76
+4 - +8	19.91	+3 - +6	24.03	+1.5 - +3	20.56
> +8	13.42	> +6	7.79	> +3	11.26

Note: After each ozone variable is constructed during the panicle formation, we first-difference the data to obtain within-county year-to-year variation of that ozone variable. Then we pool all year-to-year variation and calculate the proportion of this pooled dataset with year-to-year variation falling into a specific range, as labeled in the “Variation” columns.

Table 11: Proportions of Daily Maximum Ozone at Different Levels.

Daily Maximum (ppb)	Proportion (%)
< 60	34.61
60 - 80	28.87
80 - 100	29.25
100 - 120	4.67
$>$120	2.60

Note: This table summarizes the proportion of days during panicle formation with the daily maximum falling into certain ranges.
